# A Method to Quantify Cell-Free Fetal DNA Fraction in Maternal Plasma Using Next Generation Sequencing: Its Application in Non-Invasive Prenatal Chromosomal Aneuploidy Detection

**DOI:** 10.1371/journal.pone.0146997

**Published:** 2016-01-14

**Authors:** Xu-Ping Xu, Hai-Yan Gan, Fen-Xia Li, Qi Tian, Jun Zhang, Rong-Liang Liang, Ming Li, Xue-Xi Yang, Ying-Song Wu

**Affiliations:** 1 Institute of Antibody Engineering, School of Biotechnology, Southern Medical University, Guangzhou, China; 2 Guangzhou Darui Biotechnology Co. LTD, Guangzhou, China; 3 The Third Affiliated Hospital of Sun Yat-Sen University, Guangzhou, China; Hospital Authority, CHINA

## Abstract

**Objective:**

The fraction of circulating cell-free fetal (cff) DNA in maternal plasma is a critical parameter for aneuploidy screening with non-invasive prenatal testing, especially for those samples located in equivocal zones. We developed an approach to quantify cff DNA fractions directly with sequencing data, and increased cff DNAs by optimizing library construction procedure.

**Methods:**

Artificial DNA mixture samples (360), with known cff DNA fractions, were used to develop a method to determine cff DNA fraction through calculating the proportion of Y chromosomal unique reads, with sequencing data generated by Ion Proton. To validate our method, we investigated cff DNA fractions of 2,063 pregnant women with fetuses who were diagnosed as high risk of fetal defects. The z-score was calculated to determine aneuploidies for chromosomes 21, 18 and 13. The relationships between z-score and parameters of pregnancies were also analyzed. To improve cff DNA fractions in our samples, two groups were established as follows: in group A, the large-size DNA fragments were removed, and in group B these were retained, during library construction.

**Results:**

A method to determine cff DNA fractions was successfully developed using 360 artificial mixture samples in which cff DNA fractions were known. A strong positive correlation was found between z-score and fetal DNA fraction in the artificial mixture samples of trisomy 21, 18 and 13, as well as in clinical maternal plasma samples. There was a positive correlation between gestational age and the cff DNA fraction in the clinical samples, but no correlation for maternal age. Moreover, increased fetal DNA fractions were found in group A compared to group B.

**Conclusion:**

A relatively accurate method was developed to determine the cff DNA fraction in maternal plasma. By optimizing, we can improve cff DNA fractions in sequencing samples, which may contribute to improvements in detection rate and reliability.

## Introduction

Since the discovery of circulating free fetal DNA (cff DNA) in maternal plasma in 1997 [1], it has drawn much attention and opens up new approaches for non-invasive prenatal testing (NIPT) with a reduced risk of complications compared with invasive procedures. Applications of cff DNA include the detection of aneuploidies [2–4], diagnosis of monogenic disease [5, 6], fetal sex determination for sex-linked disorders [7] and fetal RhD status [8]. These achievements are mainly based on qualitative and quantitative analyses of cff DNA in maternal plasma, with the cff DNA fraction representing a key parameter for diagnostic algorithms in a number of these applications, especially in the detection of aneuploidies based on next generation sequencing (NGS) [9, 10]. Because the determination of chromosomal aneuploidies depends on the detection of a small increment of fetal DNA by unique sequence reads aligned to a particular chromosome, statistically expressed by z-scores, it is essential for samples located in an equivocal zone to be corrected by the cff DNA fraction. Hence, cff DNA quantitation in maternal plasma is very important in the NIPT procedure.

Currently, several approaches exist to quantify the cff DNA fraction in maternal plasma. Real-time polymerase chain reaction (PCR) is the most commonly used technology for the quantification of fetal DNA in male-bearing pregnancies, and relies on the presence of Y chromosome-specific sequences such as SRY [11–13]. To extend this method towards both male and female fetus-bearing pregnancies, a new approach has emerged that relies on the paternally-inherited fetal single nucleotide polymorphism (SNP) alleles [9, 14], as well as other new methods based on different methylation characteristics of fetal DNA and maternal DNA [15, 16]. However, all these methods require procedures and use of instruments in the laboratory that are additional to conventional methods, and are thus more expensive and time-consuming. Furthermore, basic information is required to find paternally-inherited fetal SNP allele loci with a 100% heterozygous frequency between the fetus and mother that can be used in all pregnancies, as well as the fetal specific methylation loci. Ways to quantify cff DNA fractions from the sequencing data directly without prior maternal genotype information and additional laboratory analyses are still needed.

With the advance of NGS, non-invasive fetal testing by massively parallel sequencing as a screening method for trisomies 21, 18 and 13 is very sensitive and specific and has been validated in multiple clinical trials [2, 3, 17–19]. It has been recommended that NIPT be offered to pregnant women at high risk for having a fetus with autosomal aneuploidy by several professional societies, including the American College of Obstetricians and Gynecologists (ACOG), the American College of Medical Genetics and Genomics (ACMG) and the International Society for Prenatal Diagnosis [20–22]. Although NIPT performs well, some cases are discordant with the direct karyotype. The reason for this is that circulating free DNA in the plasma of pregnant women is a mixture of placental and maternal DNA. The cff DNA is present in a wide background of maternally-derived DNAs [1, 23], and any increment in the total DNA amount (fetal and maternal) of target chromosome DNA molecules will be diluted by contributions from the pregnancy. Screening using NGS is less reliable in samples in which the proportion of cff DNA is less than 4% [24, 25]. The reliability would rise if the cff DNA fraction for sequencing could be increased. As previously reported, fetal-derived DNA molecules in maternal plasma are generally shorter than those derived from the mother [26–29]. Some researchers have also reported that the cff DNA fraction depends on the distinctive difference of size distributions of maternal and fetal DNA in maternal plasma [28]. Taking advantage of such a size difference, use of size selection during library construction of NGS may result in enrichment of cff DNA in the library for sequencing.

Consequently, the objective of this study was to develop a method to quantify fetal DNA fractions directly from NGS data, and optimize library construction procedures of NGS to increase the fraction of cff DNA. The proportional trend of cff DNA fractions among different pregnancies were analyzed by this method to increase the detection rate and reduce the false-positive rate of NIPT for trisomies 21, 18 and 13.

## Materials and Methods

### Study participants and sample collection

#### Artificial DNA mixtures

The study was approved by the clinical research ethics committee of The Third Affiliated Hospital of Sun Yat-Sen University, Guangzhou, China and all the participants signed their written informed consent approved by the ethics committee before participation in this study. DNA samples were collected from The Third Affiliated Hospital of Sun Yat-Sen University, including 112 cases of trisomy 21, 45 cases of trisomy 18, and 20 cases of trisomy 13, as well as 183 cases of euploidy. All DNA samples were extracted from early miscarriage tissues and underwent comparative genomic hybridization (CGH) for karyotype confirmation. Genomic DNA was sheared with a Covaris S2 sonicator (Covaris Inc., Woburn, MA, USA). DNA fragments of 140–200 bp were purified by XP beads (Agencourt Bioscience, Beverley, MA, USA) and quantified by Qubit^®^2.0 (Invitrogen, Life Technologies, CA, USA). Peripheral blood (50 ml) was collected into EDTA-tubes from non-pregnant healthy women aged 20–32 years. Plasma was separated by centrifugation and plasma DNA extracted using a commercial blood DNA kit (GenMag Circulating DNA from Plasma Kit, GenMag Biotech, Beijing, China) [30] following the manufacturer's instructions, quantified by Qubit^®^2.0 and stored at -80°C before use.

Artificial DNA mixture samples were prepared by adding the appropriate proportion of the fragmented male DNA fraction into the peripheral blood plasma from non-pregnant healthy women. Three kinds of fetal DNA fractions comprising 3.5% (n = 104), 5% (n = 110) and 10% (n = 146), respectively, were prepared.

#### Clinical samples

From November 2013 to May 2015, 2,063 pregnant women who were diagnosed as high risk of fetal aneuploidies by chemistry and ultrasound screening at The Third Affiliated Hospital of Sun Yat-Sen University were enrolled. 5 ml of maternal peripheral blood were collect in EDTA-containing blood tubes. Maternal peripheral blood samples were centrifuged at 1,600 x g for 10 min at 4°C [11]. The plasma portions were transferred to Eppendorf LoBind microcentrifuge tubes and centrifuged again at 16,000 x g for 10 min at 4°C to remove residual cells. Plasma aliquots were carefully transferred to fresh Eppendorf LoBind tubes. For each sample, cell-free DNA was extracted from 700 μl of plasma using the GenMag Circulating DNA from Plasma Kit (following the manufacturer's instructions) and stored at -80°C before testing.

### Library preparation

The resulting plasma-extracted DNA was used as input DNA to construct a DNA library for sequencing, separately. For each case, Ion Plus Fragment Library Kit V3, Ion Plus Fragment Library Adapters Kit (Life Technologies, USA) and AMPure XP beads were used to complete the library construction procedures consisting of end repair, adapter-ligation, amplification, and purification according to an optimized protocol similar to the Ion Xpress^™^ Plus gDNA Fragment Library Preparation User Guide (Life Technologies, USA). Two separate groups were established: for group A, 0.7x AMPure XP beads were added to the sample following end repair to remove the large size DNA; 1.1x beads were subsequently added to capture the DNA of interest. For group B, in which the large-size DNA was retained, 1.8x AMPure XP beads were added to the samples to capture DNA of all sizes in the maternal plasma samples. DNA libraries were quantified with Qubit^®^2.0. Size distributions of the libraries were verified using the Agilent High Sensitivity DNA Kit with a 2100 Bioanalyzer (Agilent Technologies, Palo Alto, CA, USA).

### Template preparation and sequencing

A mixture of 12 libraries consisting of 100 pM prepared library for each sample were performed using emulsion PCR amplification on Ion PI^™^ Ion Sphere^™^ Particles (ISPs) with the Ion OneTouch^™^ 2 Instrument (Life Technologies, USA). We enriched template-positive ISPs for up to 200 base-pair sequencing of a library using the Ion OneTouch^™^ ES Instrument. The enriched templates of 12 libraries were loaded onto one Ion PI^™^ Chip v2 and sequenced on the Ion Proton, a semiconductor sequencing platform, with an average of 3.5x sequencing coverage per nucleotide using the Ion PI^™^ Sequencing 200 Kit v3 (Life Technologies, Carlsbad, CA, USA).

### Data analysis

Statistical analysis was performed using SPSS Statistics for Windows, version 19.0. Potential differences among the three kinds of fetal DNA fraction samples were analyzed using analysis of variance (ANOVA) as appropriate, with a P value < 0.05 considered to indicate statistical significance. A paired t-test was adopted to analyze whether the cff DNA fraction increased after discarding the large-size DNA fragment. Pearson’s correlation was used to determine the relationship between cff DNA fraction and z-scores and gestational age, as well as maternal age.

## Results

### Method development

The average number of total raw reads per sample was 5 million; the mean rate of unique mapping reads was 75%. After removing low-quality and duplicate reads, a two-step correction was applied to remove the variations among bins: bin-offset correction and GC correction. The sequences were binned for each sample according to the index and mapped to the unmasked human genome sequence (hg19). The remaining unique aligned reads (except chromosome Y) were normalized to constant 100 K, then allocated to 20 K equal-sized bins and count the generated reads for each bin. The fetal aneuploidy status for chromosomes 13, 18 and 21 was determined by z-scores ($z{\text{-score}}_{i}=\left(median_{count_{i}}-median_{baseline_{i}}\right)/std_{baseline_{i}}$; -3 < z < 3, normal range) [2]. As a previous study showed that a small number of sequences in the plasma of pregnant women carrying a female fetus were wrongly aligned to the Y chromosome [31], the numbers of sequences wrongly aligned to chromosome Y in each plasma sample of pregnant woman carrying a female fetus were calculated. The proportion of unique reads aligned to chromosome Y (%*chrY*) were used to deduce the fetal DNA fractions from the chromosome Y sequences using the following equations:

$$\%chrY=\%chrY_{male}\times f-\%chrY_{female}\times \left(1-f\right)$$

$$f=\frac{\%chrY+\%chrY_{female}}{\%chrY_{male}+\%chrY_{female}}$$

where *f* is the fetal DNA fraction for each sample, %*chrY*_*female*_ is the median of proportions of sequences wrongly aligned to chromosome Y in each plasma sample from a pregnant woman carrying a female fetus (n = 1,119), %*chrY*_*male*_ is the proportion of reads aligned to chromosome Y in a plasma sample containing 100% male DNA, deduced from the 360 artificial DNA standard samples mixed with the fetal DNA. The first equation was used to calculate %*chrY*_*male*_ for each sample among the 360 artificial DNA mixture samples with fetal DNA; the median was chosen as the real %*chrY*_*male*_ to calculate the fetal DNA fractions.

### Artificial DNA mixtures samples

**Fig 1:**
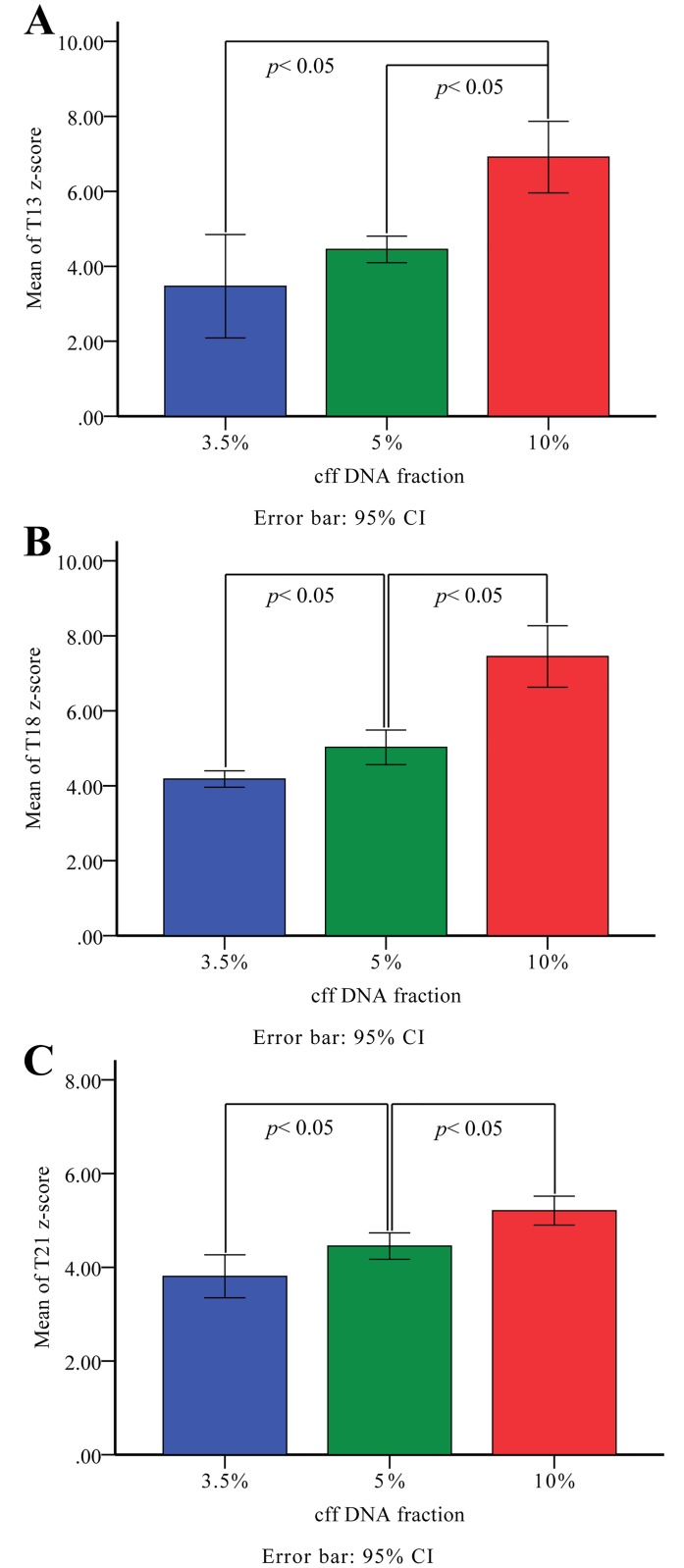
Relationships of the z-score with cff DNA fractions in artificial DNA mixture samples. The z scores of chromosome 13 (A), 18 (B) and 21 (C) are compared among three kinds of cff DNA fractions about 3.5%, 5% and 10%, respectively.

We successfully developed a method to determine the cff DNA fraction with a total of 360 artificial DNA mixture samples with varying DNA proportions (3.5%, n = 104; 5%, n = 110; 10%, n = 146) through calculating the proportion of Y chromosomal unique reads from NIPT NGS data. Moreover, we observed positive correlations between the z-score and cff DNA fractions in artificial mixture samples of trisomies 21, 18 and 13 (Fig 1).

A least significance difference (LSD) test revealed statistical significance between the z-score of chromosome 13 and cff DNA fraction in the groups with 10% and with 3.5% fetal DNA samples (*p* < 0.05), as well as the groups with 10% and 5% fetal DNA samples (*p* < 0.05).There was no significance between z-score and cff DNA fraction in the groups with 5% and 3.5% fetal DNA samples (*p* > 0.05). Results for three groups were as follows: 3.5% (mean_T13 z score_ = 3.47, 95% confidence interval [CI]: 2.09–4.85); 5% (mean_T13 z score_ = 4.45, 95% CI: 4.10–4.81), 10% (mean_T13 z score_ = 6.91, 95% [CI]: 5.96–7.87) (Fig 1A).

With respect to chromosomes 18 and 21, statistical significance was found between the z-score and cff DNA fraction in all three groups (*p* < 0.05). The mean z-scores in the groups with 3.5%, 5% and 10% cff DNA, respectively, were 4.18 (95% CI: 3.96–4.40), 5.02 (95% CI: 4.56–5.48) and 7.45 (95% CI: 6.63–8.27) for chromosome 18 (Fig 1B), and 3.81 (95% CI: 3.35–4.27), 4.45 (95% CI: 4.17–4.73) and 5.21 (95% CI: 4.90–5.52) for chromosome 21 (Fig 1C).

### Clinical maternal plasma samples

**Fig 2:**
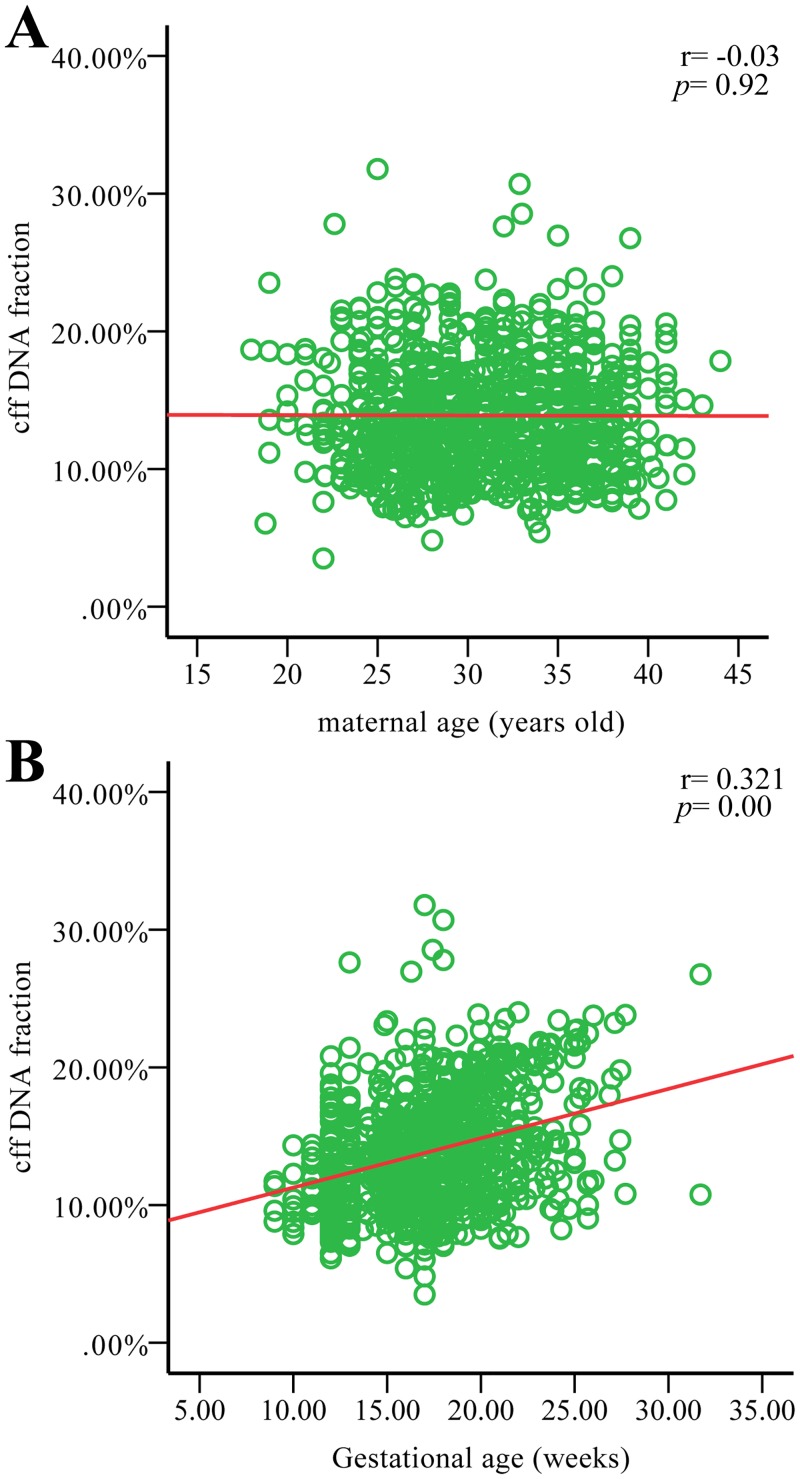
Relationships between cff DNA fractions and parameters of pregnancies. Solid lines in red show the trends between cff DNA and maternal age (A) as well as gestational age (B).

**Fig 3:**
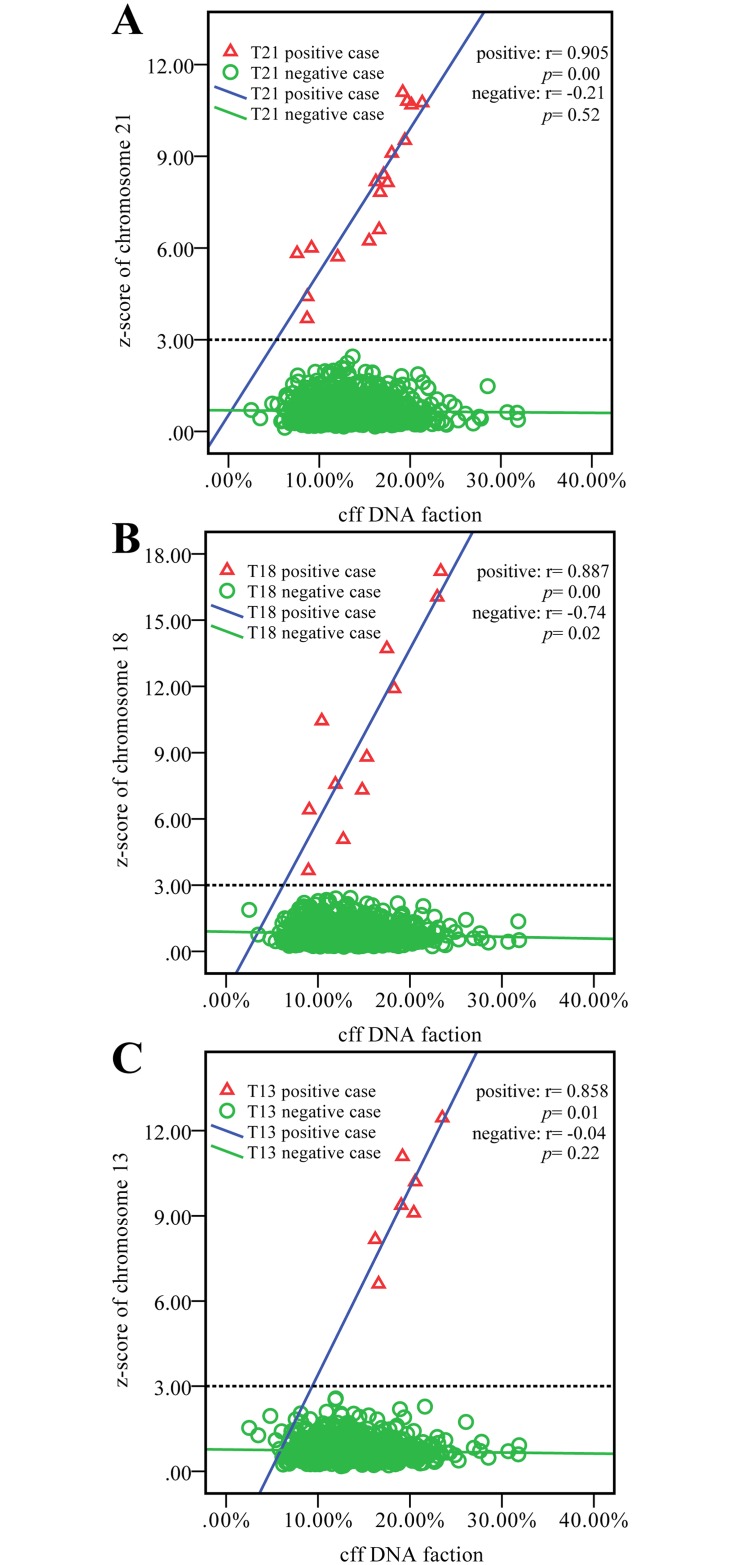
Relationships of the z-score with fetal DNA fractions in maternal plasma samples. Dash line marks the z-score cutoff of 3 for detecting trisomies of chromosome 21(A), 18(B), 13(C). Solid line markers shows the correlations between the z score and cff DNA fractions for the trisomy 21, 18, 13 cases (blue) and negative cases of chromosome 21, 18 and 13(green). Depictions are positive cases of pregnancies bearing fetuses with trisomy 21, 18, 13 (red triangles) and their negative cases (green circles).

Among the 2,063 pregnancies recruited, 944 (T13, n = 7; T18, n = 11; T21, n = 17; negative, n = 906) were carrying male fetuses (approximately 45.8%). The average cff DNA fraction was 13.89%, with a range of 4.81% to 31.88%, consistent with figures reported previously [10, 32–34].This allowed investigation of the relationship between fetal DNA fractions and z-score values and gestational age, as well as maternal age. The mean maternal age was 31 years and the mean gestational age was 17 weeks and 2 days. There was no statistical significance between the fetal DNA fractions and maternal age in our study (r = -0.03, p = 0.92) (Fig 2A). However, there was a positive correlation between fetal DNA fractions and gestational age (r = 0.321, p = 0.00) (Fig 2B). The trend appeared to represent a strong positive correlation between the z-score of chromosomes 21, 18, 13 and fetal DNA fraction in the maternal plasma when the fetus had trisomy 21 (r_T21_ = 0.905, p_T21_ = 0.00) (Fig 3A), trisomy 18 (r_T18_ = 0.887, p_T18_ = 0.00) (Fig 3B) or trisomy 13 (r_T13_ = 0.858, p_T13_ = 0.01) (Fig 3C). There was a distinct non-statistical relationship between the z-scores of chromosomes 21 and 13 and fetal DNA fractions in the negative samples of aneuploidies (r _negative (T21)_ = -0.21, p = 0.52; r _negative (T13)_ = -0.04, p = 0.22) (Fig 3A and 3C), but a negative correlation was observed between the z-scores of chromosome 18 and fetal DNA fractions (r _negative (T18)_ = -0.74, p = 0.02) (Fig 3B).

### Paired clinical maternal plasma samples

**Fig 4:**
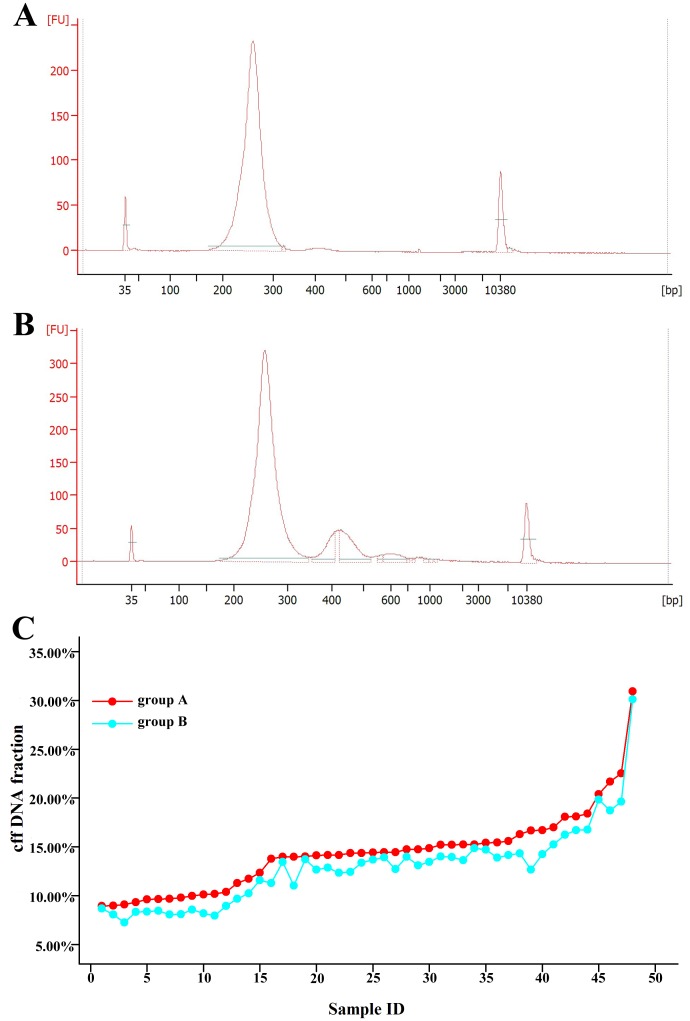
Size distributions of sequencing libraries and cff DNA fractions between group A and group B. The size distributions of DNA sequencing libraries are showed for group A (A), in which the large-size DNA fragment was discarded during library construction, and group B (B), in which it was retained. The cff DNA fractions are compared between the paired clinical samples in group A and group B (C).

A total of 48 paired clinical maternal plasma samples were collected to investigate whether an increase in the cff DNA fraction would result from discarding the large size DNA fragment during library construction. The size distributions of DNA libraries for these two groups obtained from a 2100 Bioanalyzer clearly showed that the large fragment of DNA libraries in group A (Fig 4A) was significantly less than that in group B (Fig 4B). A paired t-test showed a statistical significance between group A, in which the large-size DNA fragment was discarded, and group B, in which it was retained (mean = 1.50%, t = 13.66, p = 0.00). The cff DNA fraction increased from an average of 12.88% in group B to an average of 14.37% in group A. The cff DNA fraction of these two groups is described in detail in Fig 4C. As the fetal DNA in maternal plasma is typically shorter than 200 bp, whereas the larger-size DNA fragments mainly maternally derived DNA fragments, removing the larger-size DNA fragments with 0.7x AMPure XP beads would result in such an increase in cff DNA fraction.

## Discussion

A relatively accurate approach to infer fetal DNA fractions directly from semiconductor sequencing data of DNA in maternal plasma, without prior knowledge of fetal and parental genotype information or additional laboratory steps, was developed. In contrast to previous studies [2, 10], this method used a large number of clinical maternal plasma samples from pregnancies carrying a female fetus (n = 1,119) and artificial mixtures samples (n = 360) for which the cff DNA fractions were known, rather than using a few male adult and female fetuses as controls. Subsequently, our method has also been validated by the cff DNA fraction determinations with a large scale of clinical samples.

Comparing with other existing methodologies, such as Real-time PCR [11, 12], approach based on SNP [14] and fetal specific methylation loci [15], our method do not require additional procedures and instruments. The cff DNA fractions were directly deduced from NGS data, which do not need an extra cost and time, while the cff DNA fractions are available together with the z-scores.

As previously reported, fetal DNA is typically shorter than 200 bp, whereas a proportion of maternal DNA is larger than 200 bp in size [23, 26, 27, 35]. These observations suggest that, as a result of discarding the large size DNA fragments, the representation of shorter fetal DNA is greater, thereby increasing the cff DNA fraction. Through optimizing the procedure of NGS to increase cff DNA fractions in our sequencing samples, it may be possible to undertake aneuploidy screening via NGS at an earlier gestational age than the optimal one of 12 weeks [36]. It can also be used to increase the reliability of samples with low levels of cff DNA.

Currently, NIPT methods require a fetal DNA fraction of at least 4%. If the fetal DNA is below 4%, NIPT often fails to provide a result [24]. In our study, the relationship between cff DNA fractions and the z-score values of trisomies 21, 18 and 13 were investigated, and strongly positive correlations were observed for all trisomies. However, this trend was not observed for the negative samples of trisomies 21, 18 or 13, so the influence of the fetal DNA fraction should be considered when z-scores are used to determine chromosomal aneuploidies. Moreover, the data indicate that theoretical z-scores can be established for a series of cff DNA fractions and that NGS can be optimized to achieve enrichment of cff DNA; (i.e., by discarding the large- size DNA fragments during library construction). Therefore, removing the large-size DNA fragments may effectively help increase the detection rate and reduce the false-positive rate of NIPT for trisomies 21, 18 and 13, especially for those z-scores located in an equivocal zone with an absolute value close to 3.

Different trends of cff DNA fraction change were observed for maternal age and gestational age. Gestational age had a positive correlation with the cff DNA fraction in maternal plasma, as reported previously [10, 33]; a strong negative correlation between fetal DNA fraction and maternal weight was also reported in earlier studies [10, 32, 37]. Considering the influence of multiple factors on fetal DNA concentration, our method will contribute to a more accurate implementation of noninvasive detection methods for fetal trisomy in pregnant women. However, this method to determine fetal DNA fraction is based on the Y chromosome, so it can only be applied to pregnant women bearing a male fetus. Further research is needed to develop a method suitable for all pregnancies independent of gender.

In conclusion, we developed a method to determine fetal DNA fractions directly from sequencing data without additional experiments, which can be used as a calibration factor in the detection of fetal chromosomal aneuploidies by NGS technology. Based on this method, the procedures of NGS were modified to increase the cff DNA fraction by discarding the large size DNA fragment during library construction, which is useful to increase the reliability of samples with low levels of cff DNA and screening for aneuploidies at an earlier gestational time.
